# Characterization of CD44 variant expression in head and neck squamous cell carcinomas

**DOI:** 10.1007/s13277-013-1272-3

**Published:** 2013-10-12

**Authors:** D. Spiegelberg, G. Kuku, R. Selvaraju, M. Nestor

**Affiliations:** 1 Unit of Biomedical Radiation Sciences, Department Radiology, Oncology and Radiation Sciences, Uppsala University, Uppsala, Sweden; 2 Unit of Otolaryngology and Head & Neck Surgery, Department of Surgical Sciences, Uppsala University, Uppsala, Sweden; 3 Unit of Biomedical Radiation Sciences, Rudbeck Laboratory, Uppsala University, 751 85 Uppsala, Sweden

**Keywords:** CD44, CD44 isoforms, HNSCC, Cancer stem cells, Radioresistance, Radio-immuno targeting

## Abstract

CD44 is a complex family of molecules, associated with aggressive malignancies and cancer stem cells. However, the role of CD44 variants in tumor progression and treatment resistance is not clear. In this study, the expression of CD44 and its variants was assessed in head and neck squamous cell carcinomas (HNSCC). Furthermore, subpopulations of cells expressing high amounts of CD44 variants were identified and characterized, for e.g., cell cycle phase and radioresistance. Results revealed high and homogenous CD44 and CD44v7 expression in four cell lines and CD44v4 and CD44v6 in three cell lines. CD44v3 was highly expressed in two cell lines, whereas CD44v5, CD44v7/8, CD44v10, CD133, and CD24 demonstrated no or moderate expression. Moreover, a subpopulation of very high CD44v4 expression was identified, which is independent of cell phase, demonstrating increased proliferation and radioresistance. In cell starvation experiments designed to enrich for cancer stem cells, a large population with dramatically increased expression of CD44, CD44v3, CD44v6, and CD44v7 was formed. Expression was independent of cell phase, and cells demonstrated increased radioresistance and migration rate. Our results demonstrate that the heterogeneity of tumor cells has important clinical implications for the treatment of HNSCC and that some of the CD44 variants may be associated with increased radioresistance. Highly expressed CD44 variants could make interesting candidates for selective cancer targeting.

## Introduction

Worldwide, approximately 500,000 new cases of head and neck cancer are diagnosed each year [1, 2], and about 50 % of these patients will eventually die of the disease [3]. About 85 % of all head and neck tumors are carcinomas. This cancer type originates in the epithelium, the cells that form the lining of the mouth, nose, throat, or ear or the surface layer covering the tongue. Squamous cell carcinoma is the predominant histological type among carcinomas [4], accounting for approximately 5 % of all malignant neoplasms in Europe and USA. Even though the early stages of the disease are associated with a good prognosis, there is still a high failure rate for the more advanced stages, locally as well as at distant sites [5]. This is primarily due to late disease presentation and lack of suitable markers to detect the progression of the disease and to predict treatment outcomes. The current treatment options for head and neck squamous cell carcinoma (HNSCC) include surgery, radiotherapy, and in certain cases, chemotherapy, often in combination. However, the current treatment modalities often fail in the therapy of advanced disease, as they offer no satisfactory means to locate or treat metastases selectively without harming adjacent tissues [6]. An effective adjuvant systemic therapy for minimal residual disease is therefore needed to improve the survival rate and improve quality of life for this patient category.

Selective targeting of cancer cells based on cell surface molecules may enable personalized diagnosis and treatment, which promises to lower adverse effects and increase treatment efficacy, improving patient survival and quality of life. Tumors are, however, highly heterogeneous, and it is important to identify especially aggressive, chemo-, and/or radioresistant tumor subpopulations for effective treatments [7].

One interesting target is CD44 and its isoforms. CD44 is a complex family of molecules, produced from one gene by alternative splicing and posttranslational modifications. To date, dozens of different CD44 isoforms have been discovered; the most common one is the standard CD44 (CD44s), in which exon 5 is spliced directly to exon 16, skipping the entire variant exon sequence [8, 9]. Exons 6–15 are variable exons, alternatively spliced in insertion site between 5 and 16, generating various isoforms of the molecule. These variable exons are identified as v1–v10, respectively. The isoforms mostly differ in size and conformation of the extracellular domain, which determines what binding partner the receptor interacts with. Accordingly, the CD44 family is involved in cell–cell and cell–matrix interactions and signal transduction [10]. They are not only essential to multiple biological functions of normal cells but also crucial to many tumor cell activities, as malignant cells often express unique patterns of CD44 isoforms [11].

Several studies have found CD44 to have a role in impaired prognosis in many malignant tumors, such as hepatic carcinoma, lung carcinoma, breast carcinoma, melanoma, gastric carcinoma, and HNSCC. Moreover, overexpression of CD44 in tumors has been shown to predict an increased resistance to chemotherapy and radiation and an increased risk of local recurrence [12]. Furthermore, several studies describe CD44 to be a cancer stem cell marker for various malignancies [13, 14]. This makes it a highly interesting candidate for selective cancer targeting. However, antibody-sorting assays of CD44 used in these studies do not only select cells expressing the standard CD44s molecule, but this sorted population may also contain any CD44 splice variants. If one or several CD44 splice variants are found to be associated with cancer progression or treatment resistance, the possibility of targeting such a population will increase immensely, since these isoforms are not as abundantly expressed in the normal tissue as the standard CD44s molecule.

Besides CD44, a number of CD markers have been identified as cancer stem cell markers in several solid tumors. The glycoprotein CD133, also known as prominin1, is a stem cell surface antigen in brain, colorectal, and prostate cancer [15]. Another cell surface marker is the single-chain sialoglycoprotein CD24, which is associated with cancer stem cell characteristics in colorectal and pancreatic cancer, while head and neck cancer cells and breast cancer cells with CD44^+^CD24^−/low^ expression are highly tumorigenic [16–18].

The scope of this study was to study the expression of potential cancer stem cell markers CD133, CD24, and CD44, with focus on CD44 exon variants in head and neck squamous cell carcinoma, in order to find attractive molecular targets for selective cancer targeting. The extent of variant expression was first studied in several HNSCC cell lines, and possible subpopulations of cells expressing high amounts of one or several variants were then identified. The role of these subpopulations with regards to, e.g., cell cycle phase, migration rate, proliferation, and radioresistance was then assessed.

## Material and methods

### Cell lines and culture conditions

#### Standard culture conditions

The HNSCC cell lines SCC-25 (derived from SCC of the tongue), obtained from American Type Culture Collection, and H314 (floor of mouth, STNMP stage II, moderately differentiated, node positive tumor), obtained from European Collection of Cell Cultures, were cultured in a 1:1 mixture of Ham’s F12 and Dulbecco’s modified Eagle medium (DMEM), supplemented with 10 % fetal calf serum, 2 mM l-glutamine, and antibiotics (100 IU penicillin and 100 μg/ml streptomycin). The HNSCC cell lines UT-SCC7 (metastasis/neck, temporal skin, tumor grade 2, TNM at diagnosis T1N0M0) and UT-SCC12 (primary, skin of nose, tumor grade 1, TNM at diagnosis T2N0M0) (kindly provided by Dr. R. Grenman, Turku University Central Hospital, Finland) were cultured in DMEM, 5 ml nonessential amino acids, and supplements. Medium with supplements is referred to as “complete medium” in this article. Cells were incubated at 37 °C in an atmosphere containing humidified air with 5 % CO_2_.

#### Serum starvation

Cell growth under serum starvation was performed as in the study of Okamoto et al. [19], previously shown to increase the fraction of cancer stem-like cells. For serum starvation experiments, SCC-25 and UT-SCC7 cells were grown in a cell culture medium containing 1 % fetal bovine serum (Sigma-Aldrich) and epidermal growth factor (EGF; Calbiochem, Germany) and basic fibroblast growth factor (bFGF; Calbiochem; 20 ng/ml each) for up to 5 months. Analysis was performed by flow cytometry (see below).

### Antigen expression, cell sorting, and cell cycle analysis by flow cytometry

#### Antigen expression

The HNSCC cells SCC-25, UT-SCC7, UT-SCC12, and H314 were harvested by nonenzymatic cell dissociation solution (Sigma-Aldrich, St. Louis, USA) and washed three times in phosphate-buffered saline (PBS) containing 0.5 % bovine serum albumin (BSA). Then, cells were incubated for 10 min with labeled antibodies [phycoerythrin (PE)-conjugated anti-CD133 1:100, allophycocyanin (APC)-conjugated anti-CD44 1:1,000, and fluorescein isothiocyanate (FITC)-conjugated anti-CD24 1:100 (eBioscience, San Diego, USA); FITC-conjugated mouse antihuman v4, v5, v6, v7, v7/8, and v10 (all from AbD Serotec, Kidlington, UK); and PE-conjugated mouse antihuman CD44v3 and CD44v4/5 (R&D Systems, Minneapolis, USA)]. Respective immunoglobulin G (IgG) isotype-matched controls, APC-conjugated rat IgG2b 1:1,000, PE-conjugated mouse IgG1 1:100, and FITC-conjugated mouse IgG1 1:100 (AbD Serotec, Kidlington, UK; eBioscience, San Diego, USA; R&D Systems, Minneapolis, USA), were used as negative controls.

Cells were washed three times with PBS with 0.5 % BSA and analyzed with the BD LSRII SORP (Becton Dickinson Biosciences, San Jose, USA) flow cytometer. Debris and apoptotic cells were stained with propidium iodide (Sigma-Aldrich) and excluded from analysis. The experiments were repeated at least three times.

#### Cell cycle progression

To determine if the CD44 variant expression pattern was consistent through the cell cycle, cell cycle studies were performed. To first ensure that the cells progress through the cell cycle in a synchronous manner, SCC-25, UT-SCC7, and UT-SCC12 were grown in low serum (0.5 %) condition. Depending on the doubling time of the HNSCC cell lines, cells were maintained in low serum media for 12–48 h. After quiescence in G0 phase, cells were released by adding a complete culture medium and harvested at different time points.

For analysis of CD44 and CD44 variant expression through the different phases of the cell cycle, cells were detached with nonenzymatic cell dissociation solution (Sigma-Aldrich, Stockholm, Sweden), washed in PBS with 0.5 % BSA, and fixed in ice-cold 70 % ethanol. Cells were treated with RNase (Sigma-Aldrich) and stained for 30 min with propidium iodide solution with 0.1 % NP-40 (Sigma-Aldrich) and 10 min with the antibodies listed earlier, followed by analysis with the BD LSRII SORP (Becton Dickinson Biosciences, San Jose, USA) flow cytometer. Un-synchronized cells were stained as above and used as a control. The percentages of cells in the different cell cycle phases (G0/G1, S, or G2/M phase) were determined using FACSDiva (Becton Dickinson Biosciences, San Jose, USA) and ModFit LT (Verity Software House, Tosham, ME, USA) software. Each experiment was repeated at least three times.

#### Fluorescence-activated cell sorting

For the sorting experiments, UT-SCC7 cells were prepared as described earlier in antigen expression and evaluated in a FACSVantage SE DiVa (Becton Dickinson Biosciences, San Jose, USA). Populations were gated, and the cell populations expressing the marker to high extent and low extent were separated.

### Cell proliferation, radiation resistance, and migration studies

#### Cell proliferation and radiation resistance

Proliferation- and radiation-mediated effects in subpopulations or starved cells were studied using clonogenic survival assays on SCC-25 or UT-SCC7 cells.

For clonogenic characterizations of starved cells, untreated and serum starved SCC-25 cells were grown for 4–16 weeks. Clonogenic survival assays were performed in triplicates. Here, a defined amount of cells (200 to 10,000, depending on radiation dose) was pre-plated into 25-cm^2^ tissue culture flasks with 8 ml complete medium. After 24 h, cells were exposed to external beam radiation using a ^137^Cs source (Best Theratronics Gammacell® 40 Exactor, Springfield, USA), irradiated with 0, 2, 4, 6, and 8 Gy. After an incubation period of approximately 10–14 days, cells were washed in PBS and stained with crystal violet. Colonies of greater than 50 cells per colony were counted manually. Plating efficiency (PE) (number of colonies formed/number of colonies seeded in the control) and the survival fraction (SF) (number of colonies formed after treatment/number of cells seeded × PE) were calculated. Linear quadratic curve fit (*S* = exp (−α*D* − *βD*_2_), where *D* = radiation dose in Gray, and *α* and *β* are fitting parameters) was calculated by using the GraphPad Prism 5 software (San Diego, CA, USA). Significance testing was made using two-tailed Student’s *t* test and was considered significant if *P* < 0.05. Experiments were repeated at least three times.

For subpopulation analysis, defined subpopulations of UT-SCC7 cells were sorted using FACSVantage SE DiVa as described above, and a defined amount of cells was plated in triplicates. After 24 h, cells were exposed to 2 Gy external beam radiation using a ^137^Cs source (Best Theratronics Gammacell® 40 Exactor, Springfield, USA). After an incubation period of approximately 10–14 days, cells were washed in PBS, stained with crystal violet, and analyzed as described above.

#### Cell migration

The migratory activity of starved cells was studied using the scratch assay, as previously reported [20], on SCC-25 cells. Briefly, cells were grown in triplicates under normal and starvation growth conditions in six-well plates for 4–6 weeks (see above). Cells were washed and incubated with corresponding cell medium. Then, a narrow area on the confluent cell monolayers was scratched off with a p200 pipette tip. Cells were allowed to migrate for 8–12 h, images from the same areas were obtained using the Nikon D3000 Digital Camera mounted on an inverted microscope. The images were analyzed with the ImageJ software (National Institutes of Health, Bethesda, Maryland). Significance testing was made using two-tailed Student’s *t* test and was considered significant if *P* < 0.05. Experiments were repeated three times.

## Results

### Characterizations in standard culturing conditions

#### General antigen expression assessed by flow cytometry

**Fig. 1:**
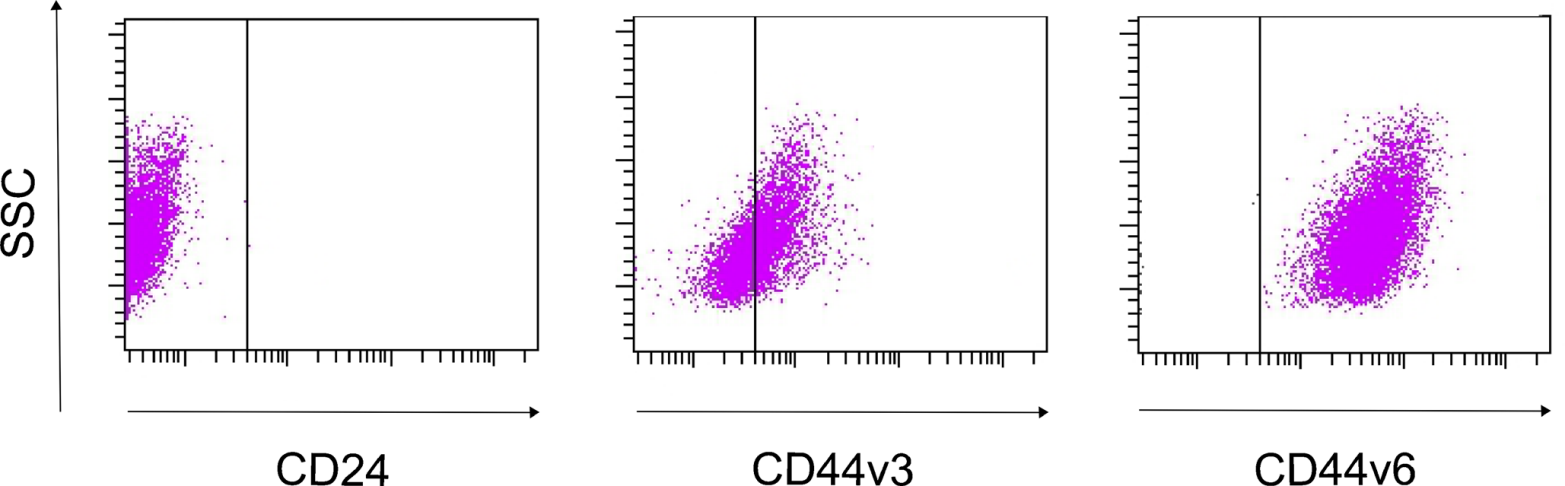
Example of FACS analysis of three markers differently expressed in the HNSCC cell line SCC-25. **a** Example of a marker, CD24, which displayed no or low expression. **b** Example of a marker, CD44v3, which displayed moderate expression. **c** Example of a marker, CD44v6, which displayed a high expression

The expression pattern of all investigated antigens is summarized in Table 1. Examples of the expression pattern after analysis with flow cytometry can be seen in Fig. 1. All four investigated HNSCC cell lines showed no or low expression patterns for CD24. CD133 was moderately expressed in three of four cell lines, whereas all cell lines were positive for CD44 expression. Since the staining for CD44 only gives information about the total expression of CD44, but not for the ratios between different splice variants and the CD44 standard form, the expression pattern of CD44 exon isoforms was also investigated. The extent of expression varied only slightly between the cell lines. CD44v4, CD44v6, and CD44v7 were highly expressed in all tested HNSCC cell lines, whereas CD44v5, CD44v8, and CD44v10 occurred to lesser extent. The CD44v3 level in UT-SCC7 and UT-SCC12 was high, while SCC-25 and H314 moderately expressed this marker (Table 1).

**Table 1:** Expression pattern of CD44, CD44 exon variants, CD133, and CD24. The expression levels were measured by flow cytometry and graded on a relative scale to isotope control (expression baseline)

Cell line	Expression of the marker									
	CD133	CD24	CD44	v3	v4	v5	v6	v7	v7/8	v10
UT-SCC7	+	−	++	++	++	+	++	++	−	+
UT-SCC12	+	−	++	++	++	+	++	++	−	+
SCC-25	+	−	++	+	++	−	++	++	−	+/−
H314	+/−	−	++	+	+	−	+	++	−	−

– low or no expression; + moderate expression; ++ high expression; *N* = 2–6

#### Antigen subpopulations assessed by flow cytometry

**Fig. 2:**
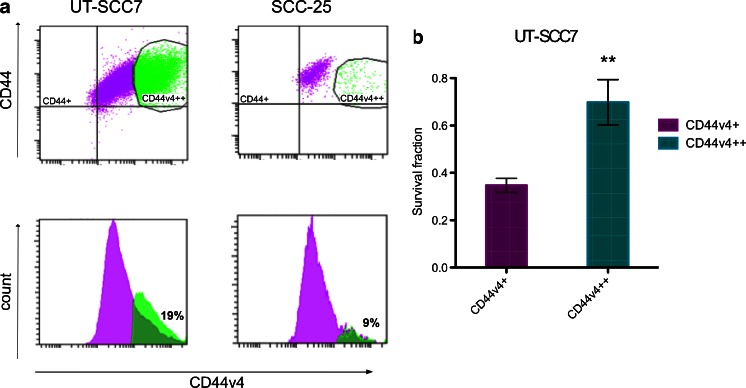
**a** Dot plot and histogram of FACS analyzed UTSCC-7 and SCC-25 cells. All cell lines were positive for CD44v4 (*purple population*). Additionally, a distinct subpopulation with CD44v4 overexpressing cells was found (*green population*). **b** Clonogenic survival assay of FACS-sorted UT-SCC7 cells. CD44v4-positive and CD44v4 overexpression cells treated with a radiation dose of 2 Gy. CD44v4+ and CD44v4++ groups are normalized for plating efficiency (unirradiated controls). Analyses of variance was made using Student’s *t* test and was considered significant if *P* < 0.05. The *error bars* represent standard deviation (SD), *N* = 3

A homogenous expression with no clear subpopulations was seen for all assessed markers in standard culturing conditions, except for CD44v4. This interesting CD44v4 population consisting of highly expressing cells (CD44v4++) was found in the investigated cell lines. These cells differed markedly from the bulk of all HNSCC tumor cells and appeared as a clear subpopulation (Fig. 2a). This subpopulation was about 19 % in UT-SCC7 cells and 7, 9, and 10 % in UT-SCC12 cells, SCC-25 cells, and H314 cells, respectively. Furthermore, this expression seemed to be cell cycle independent, as cell cycle studies confirmed unchanged expression of CD44 and CD44v4 throughout the different cell cycle phases (data not shown).

#### Antigen subpopulations assessed by clonogenic assays

Next, we examined proliferation and sensitivity to radiation of the subpopulation of highly CD44v4-expressing cells in comparison to cells with the lesser extent of CD44v4 in the cell line with the largest CD44v4++ subpopulation (UT-SCC7, Fig. 2b). Highly expressing cells were isolated from the bulk of tumor cells and cultured separately. Immediately after separation, clonogenic survival assays were performed on the separated populations. The plating efficiency of CD44v4++ cells was increased by approximately 20 % compared to CD44v4+ cells. Moreover, v4++ cells were extensively more resistant to radiation treatment than CD44v4+ cells. More than 70 % of CD44v4++ cells kept their colony-forming ability after a radiation dose of 2 Gy, while less than half (around 34 %) of CD44v4+ cells were able to grow into a colony.

### Characterizations in serum starvation conditions

#### Morphology and antigen expression assessed by flow cytometry

**Fig. 3:**
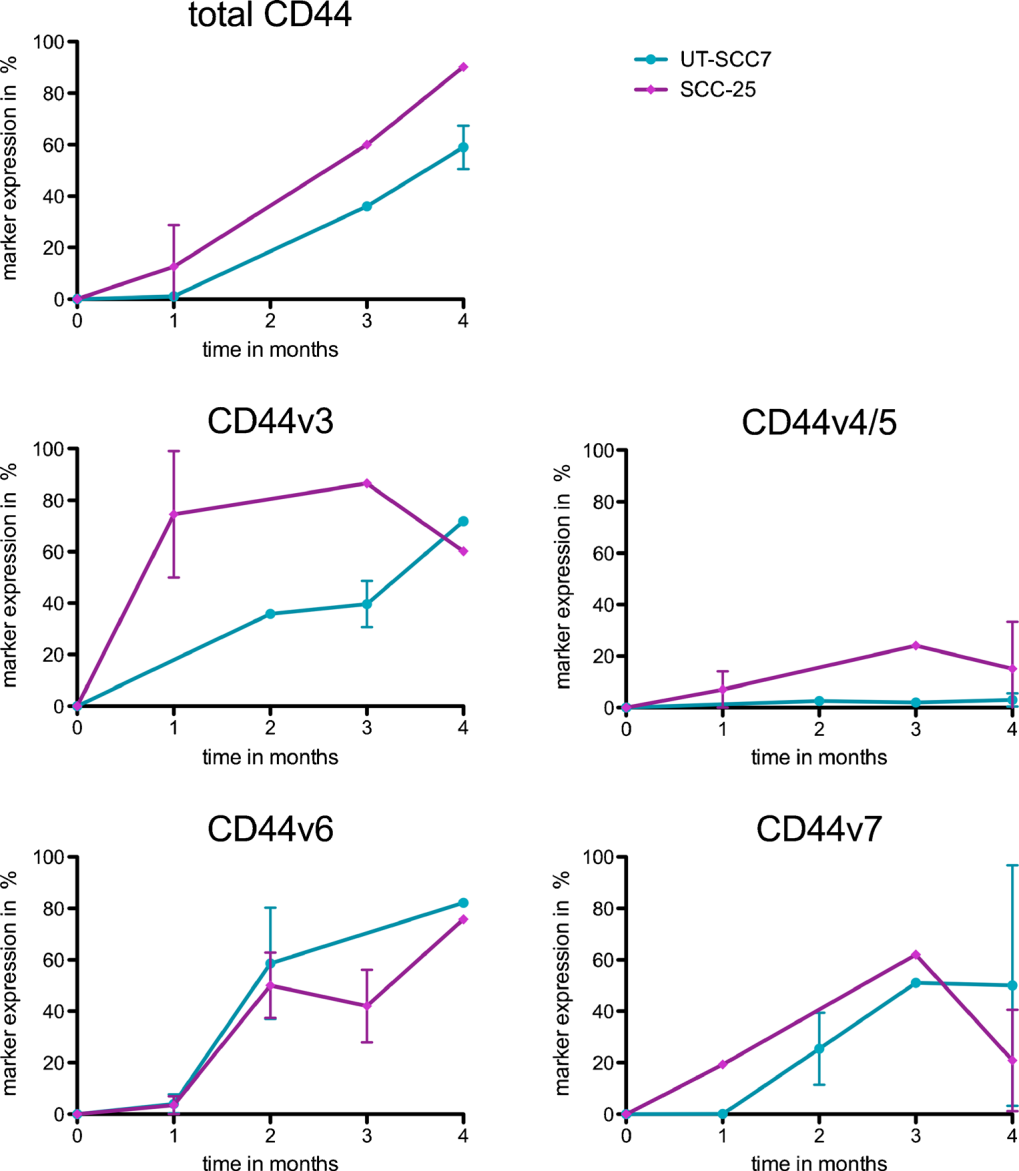
FACS analysis of UT-SCC7 and SCC-25 cells. Change of CD44, v3, v4/5, v6, and v7 marker expression under starvation condition, normalized to their controls. Marker expression of non-starved cells is set to 0 %. *Error bars* = SD, *N* ≥ 2–4

SCC-25 and UT-SCC7 cultured under low serum conditions underwent clear morphological changes, while cells under normal conditions did not. Starved cells grew slowly and formed tight cell aggregates. Flow cytometry analysis showed that both starved and non-starved cells were positive for CD44 expression. However, the amount of highly CD44-expressing cells had increased in the cell populations under starvation conditions. After 4 months of starvation, an average of 59 % for UT-SCC7 and 90 % of SCC-25 had at least doubled their CD44 expression (Fig. 3). Even though the expression pattern for CD44 changed, the CD44 variants v5, v7/8, and v10 did not. However, a substantial increase of CD44, CD44v3, CD44v6, and CD44v7 and a slight increase for CD44v4/5 were found in both cell lines. After serum starvation of 4 months, the CD44 expression had increased with 90 % in SCC-25 and with 59 % in UT-SCC7 cells. UT-SCC7 control cells, cultured under normal conditions, were positive for CD44v3, CD44v6, and CD44v7, but only a minority of cells (3 % for CD44v3, 4 % for CD44v6, and 2 % for CD44v7) did overexpress the marker. In contrast, more than 70 % of the serum starved cells overexpressed variant v3, and more than 80 and 50 %, v6 and v7, respectively, after 4 months of treatment (Fig. 3). Similar results were obtained for SCC-25 cells. Here, the CD44v4/5 variant increased with about 15 %; v3 expression, with 60 %; v6, with 75 %; and v7, with about 21 % (Fig. 3).

Furthermore, to determine if the CD44 variant expression pattern was consistent through the cell cycle, cell cycle studies were performed. Neither CD44 nor any of the other CD44 exon isoforms appeared to change noticeably throughout the cell cycle (data not shown).

#### Radiation resistance and migration of starved cells assessed by clonogenic and scratch assays

**Fig. 4:**
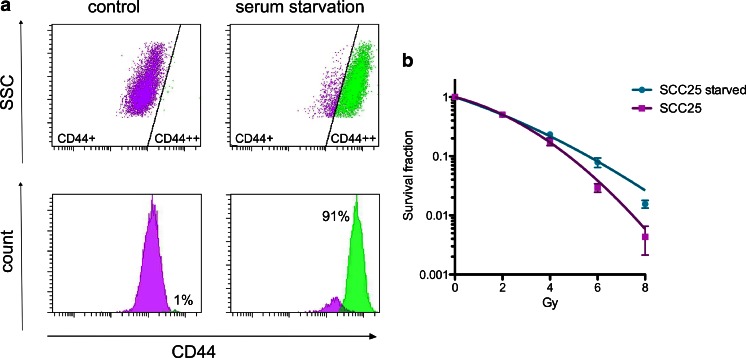
**a** FACS analysis of SCC-25 cells under normal growth condition and after serum starvation of 4 months (1 % FBS, 20 ng/ml EGF and bFGF) for CD44. Serum starvation increased the amount of cells highly expressing the marker. **b** Clonogenic survival assay of SCC-25 cells under normal growth condition and after serum starvation of 4 months (1 % FBS, 20 ng/ml EGF and bFGF). Normal SCC-25 cells are more sensitive to radiation than previously starved SCC-25 cells. Survival data was fit to a linear quadratic curve. The *error bars* represent SD. *N* ≥ 9

To examine if previously starved and, hence, CD44 positive (Fig. 4a) cells differed in their ability to survive radiation treatment, clonogenic survival assays were performed on SCC-25 cells (Fig. 4b). Previously starved cells were clearly more resistant to radiation than normal cells, statistically significant (*P* < 0.05) for 4, 6, and 8 Gy. Approximately 25 % of starved cells survived a radiation dose of 4 Gy, whereas only 12 % of non-starved cells could form a colony after the same treatment. The difference was even more pronounced at a radiation dose of 6 and 8 Gy. Migration analysis was also assessed for SCC-25 cells starved for 4–6 weeks. Starved cells migrated significantly (*P* < 0.001) faster than non-starved cells, demonstrating a migration rate of 0.045 ± 0.002 mm/h compared to 0.017 ± 0.003 mm/h for non-starved cells.

## Discussion

The purpose of this study was to examine the expression of the cancer stem cell markers CD133, CD24, CD44, and CD44 variants, in head and neck squamous cell carcinoma. The aim was to find suitable molecular targets for radio-immunodiagnostics and therapy and to clarify if any of the CD44 splice variants may be associated with radioresistance. Furthermore, since serum-starved conditions have been shown to increase the amount of CD44-positive cancer stem-like cells [19], we wanted to elucidate if this increase was also true for any of the CD44 splice variants. It is important to emphasize that most antibody-mediated expression analyses of CD44, such as immunohistochemistry (IHC) or FACS analyses, will not distinguish between standard CD44s or any of the CD44 isoforms, as they all share the same standard region. As a majority of studies have not taken into consideration that such CD44 analyses will detect a collection of isoforms, it is not surprising that no consensus opinion has been reached on the role of CD44 and its isoforms in tumor progression and cancer stem cells. Hence, it is yet not known if one or several CD44 isoforms can be utilized as markers for radioresistance or even cancer stem cell markers.

Whereas some tumors such as gliomas exclusively express CD44s, other neoplasms including gastrointestinal cancer, bladder cancer, uterine cervical cancer, breast cancer, and HNSCC also express CD44 variants. This may be due to a loss of CD44 splice control mechanisms in malignant tumors. Overexpression of several CD44 isoforms has been associated with tumor progression, suggesting that these isoforms may have unique signaling properties. Hence, CD44, particularly its variants, may be used as diagnostic or prognostic markers of at least some human malignant diseases [21], even enabling specific targeting of aggressive, resistant, or regrowing subpopulations. However, not much is known today about the role of most of these variants in malignancies, cancer stem cells, and HNSCC.

In this study, flow cytometry analysis was first used to assess the extent of CD133, CD24, and CD44 variant expression in four HNSCC cell lines. Our results demonstrated high and homogenous expression of CD44 and CD44v7 in four cell lines and CD44v4 and CD44v6 in three of the cell lines. CD44v3 was highly expressed in two cell lines, whereas CD44v5, CD44v7/8, CD133, and CD24 demonstrated no or low expression.

CD44 isoforms containing exon v3 have recently drawn attention since this exon includes a growth factor-binding site (such as HGF, bFGF, and HB-EGF), and this domain seems to be critical to the metastasis phenotype [22, 23]. Studies suggest that CD44v3-containing isoforms are overexpressed at the mRNA level in HNSCC tissues compared to normal controls and may be associated with HNSCC cell growth [23]. CD44v3-containing isoforms have also been associated with HNSCC migration, clonal formation, cisplatin resistance, and matrix metalloproteinase activity and have been identified in lymph node metastasis [24, 25]. CD44v3 isoforms may, therefore, be effective tumor markers and targets for HNSCC therapy. It is interesting to note that in our study, CD44v3 was highly expressed in the two SCC cell lines derived from the skin (UT-SCC7 and UT-SCC12), whereas the SCC cell lines derived from the tongue and floor of mouth (SCC-25 and H314, respectively) demonstrated a moderate CD44v3 expression. A similar difference could also be seen for CD44v5 and CD44v10 expression (moderate versus no/low expression). However, a much larger panel of cell lines, preferably complemented with expression studies in patient tumor tissue, would be needed in order to draw any conclusions on CD44 variant expression correlated to tumor origin or tumor progression. Furthermore, since cell lines were cultured according to provider instructions, UT-SCC7 and UT-SCC12 cells were cultured in a slightly different medium than SCC-25 and H314, further complicating such comparisons.

Expression of CD44v4 has been associated with metastasis in two studies of breast cancer cells and esophageal squamous cell carcinoma, where it was shown to be a major E-selectin ligand in facilitating tumor cell migration across endothelial monolayers, mediating breast cancer cell transendothelial metastasis [26]. Furthermore, expression of CD44v4/5 in esophageal squamous cell carcinoma has been linked to infiltration and metastasis [27]. However, not much is known about the role of CD44v4 in HNSCC. It is, therefore, exciting that such a clear overexpression of CD44v4 was found in our assessed cell lines, indicating that CD44v4 has potential to be used as a novel tumor marker. An interesting subpopulation (>7 %) of very high antigen expression was identified in all four cell lines in our study. This subpopulation displayed increased proliferation and radiation resistance after external beam irradiation with 2 Gy. One hypothesis of these results could be that CD44v4 is associated with a more dormant phase. However, the CD44v4 expression was shown to be independent of cell phase, making this theory less likely. Previous studies have demonstrated a connection between radiation and E-selectin, suggesting that ionizing radiation induces E-selectin protein expression in human endothelial cells and, therefore, increasing tumor cell adhesion [28, 29]. Thus, an alternative explanation for the CD44v4-associated radiation resistance may be that CD44v4 functions as an E-selectin ligand, mediating increased adhesion properties at radiation exposure. We conclude from these results that not only CD44s but also CD44v4 seems to be associated with radioresistance and that CD44v4 may be an interesting tumor marker to be further studied.

CD44v6 is currently the most established tumor antigen among the CD44 splice variants. The large difference in expression between healthy and malignant cells makes the CD44v6 antigen an attractive target for radio-immunodiagnostics and therapy. The anti-CD44v6 antibody U36 has been extensively studied in patients with primary HNSCC and lymph node metastases [30] and been evaluated with encouraging results in clinical trials using ^186^Re as a therapeutic radionuclide [31]. CD44v6 is known to be expressed in many types of human cancer, including HNSCC. It has been shown to function as a co-receptor of the c-Met protein [32] and has been suggested to be involved in tumor formation, tumor cell invasion, and metastasis formation [30]. One distinctive CD44 isoform, so-called pMeta-2 and containing the various exons 6 and 7, has drawn attention since it is associated with cancer aggressiveness and metastatic behavior [33]. It is, therefore, not surprising that we found a high CD44v6 expression in three of our assessed cell lines, verifying the established position of CD44v6 as a marker for HNSCC. Even more interesting, CD44v6 and CD44v7 and, to some extent, CD44v3 were also shown to be highly relevant in cell starvation experiments.

In these starvation experiments, designed to increase the population of cancer stem-like cells, we were able to enrich the proportion of CD44-positive cells by culturing in low serum containing cell medium with EGF and bFGF. This is in line with previous results by Okamoto et al. [19]. The properties of serum starved cells with regards to, e.g., cell cycle phase, proliferation, migration, and radioresistance were evaluated. So far, CD44 expression on cancer cells has been shown to regulate multiple aspects of cancer cell phenotypes, modulating tumor proliferation, migration, invasion, and angiogenesis. We found that serum-starved CD44-enriched cells had an increased radioresistance and migration rate compared to non-starved cells, indicating an important role for clinical applications. While CD44 has been defined as a cancer stem cell marker in many tumor entities, it remains to be explored whether these cancer stem cells preferentially express CD44s or any CD44 splice variants [34]. Further analysis of highly CD44-expressing cells demonstrated that the antigen expression for CD44 variants was practically unchanged for the analyzed variants, except for CD44v3, CD44v6, and CD44v7 (Fig. 3), as previously mentioned. This is in line with a study on colorectal adenocarcinoma by Bánky et al., where high expression levels of CD44v3 and CD44v6 were found to be characteristic to metastatically potent tumor cells, and CD44 variant isoforms were described to act as “metastasis genes” via tumor microenvironment-driven shifts in v3 and v6 expressions [35]. In our study, a large subpopulation of dramatically increased expression was formed for these isoforms. Moreover, we noticed that the morphology of the starved cells was altered, which renders fewer cells growing as a monolayer and a larger proportion of cells growing three-dimensionally. We speculate that these conformational changes might in part be caused by the increased CD44 expression. From these results, we conclude that the previously found CD44 increase in “cancer stem-like cells” may also consist of several splice variants, primarily CD44v3, CD44v6, and CD44v7, and that these splice variants should be further assessed concerning their connection to cancer stem cell-like properties. It is, however, important to note that these results are based on a limited selection of cell lines. Clearly, more studies are needed to further validate this approach, especially by investigating CD44 expression levels in various patient tissue biopsies. Even though IHC is commonly used for the analysis of patient biopsies, it is a comparatively blunt method and might be unable to satisfactorily distinguish between moderately and highly CD44 variant-expressing cells within the same biopsy. It is, therefore, likely that alternative methods such as FISH or RT-PCR will have to be used as a complement. Another important aspect is the molecular mechanism underlying the radioresistance of highly CD44 variant-expressing cells, which warrants further investigation.

In conclusion, we have identified several markers that are highly expressed in cultured HNSCC cells, and we have also identified splice variants associated with increased radioresistance and migration rate. Highly expressed variants of CD44 may also be a more specific marker than standard CD44s, which is present to high extent also in normal tissue. Our results demonstrate that the heterogeneity of tumor cells has important clinical implications for the treatment of HNSCC. In the future, CD44 variants may be used as prognostic markers or therapeutic targets. By characterizing these novel molecular tumor markers, we hope to advance our understanding of the molecular and biological mechanisms involved in carcinogenesis and tumor progression and to find unique CD44 isoform and expression fingerprints of the varying cell types present in tumors. This could enable the specific targeting of aggressive, resistant, and regrowing subpopulations and provide more sensitive and specific methods for identifying and treating head and neck cancer in the future, which, in turn, is likely to manifest as increased treatment success rates and posttreatment lifetime expectancies of HNSCC patients.
